# Chronic Elbow Nodules Caused by *Fomitiporella* Fungus in Immunocompetent Patients, United States 

**DOI:** 10.3201/eid3209.260263

**Published:** 2026-09

**Authors:** Giannoula S. Tansarli, Bicong Wu, Stephen J. Salipante, Brad T. Cookson

**Affiliations:** University of Washington School of Medicine, Seattle, Washington, USA (G.S. Tansarli, S.J. Salipante, B.T. Cookson); Sanford Laboratory Amber Valley, Fargo, North Dakota, USA (B. Wu)

**Keywords:** *Fomitiporella*, fungi, fungal infection, PCR, molecular diagnostics, sequencing, subcutaneous infection, basidiomycetes, United States

## Abstract

We report 2 cases of chronic subcutaneous infection in immunocompetent persons caused by *Fomitiporella* spp. basidiomycetes. Both patients recovered after surgical excision without antifungal therapy. We confirmed the cause of infection using broad-range fungal PCR from formalin-fixed tissue, highlighting the value of molecular diagnostics for detecting rare environmental fungi.

Although systemic fungal infections are most often associated with immunocompromised patients, several well-described fungal diseases, such as chromoblastomycosis, mycetoma, and sporotrichosis, cause localized skin and subcutaneous infections in immunocompetent persons. The range of environmental fungi known to cause localized infections in immunocompetent persons is expanding (*1*). Such infections often follow a prolonged, indolent course and remain confined to specific organs, such as the skin and subcutaneous tissues. Subcutaneous fungal infections involve deeper layers of the skin and underlying tissues and usually result from infection by low-virulence organisms (*2*). Acquisition of fungi involved in localized infections can occur via environmental exposure, inhalation followed by localized disease, or traumatic inoculation (*1*–*3*). Traumatic inoculation is the most common portal of entry in cutaneous and subcutaneous fungal infections, frequently involving exposure to contaminated vegetation, wood, or soil.

Among the diverse fungi that cause opportunistic or localized disease, many members of the phylum Basidiomycota (basidiomycetes), including yeasts such as *Cryptococcus* and *Malassezia* as well as filamentous fungi, are environmental organisms that are rarely implicated in human disease. Filamentous basidiomycetes present a particular diagnostic challenge to clinical laboratories because they often fail to sporulate in culture; they might be mistakenly classified within the broad category of hyaline molds because they are often indistinguishable by conventional morphologic methods (*4*,*5*). Since the early 2000s, molecular techniques have increasingly been used to improve recognition and classification of such organisms (*6*); specific filamentous basidiomycetes have been implicated in hypersensitivity syndromes, chronic cough, allergic bronchopulmonary disease, sinusitis, and, rarely, invasive infections (*5*,*7*,*8*). However, the pathogenic potential for many genera within Basidiomycota remains unknown.

*Fomitiporella* is a genus of filamentous basidiomycetes comprising wood-decaying fungi (*9*) with no well-established role in human disease. Here, we report 2 cases of localized *Fomitiporella* infection in immunocompetent patients in the United States, both diagnosed using broad-range fungal PCR.

## The Study

Case 1 was in a 46-year-old man in North Dakota, USA, who immigrated from Liberia in 2012. When he sought care in 2025, he had a 2-year history of localized pain, discomfort, and restricted movement over his right olecranon bursa. He reported no injury to the area, fever, night sweats, weight loss, or respiratory symptoms. His medical history was unremarkable, and he was taking no prescription medications. Magnetic resonance imaging demonstrated olecranon bursitis. After orthopedic evaluation, surgical excision of the bursa was conducted. Postoperative recovery was uncomplicated; sensation, motor strength, and range of motion were fully preserved.

**Figure 1:**
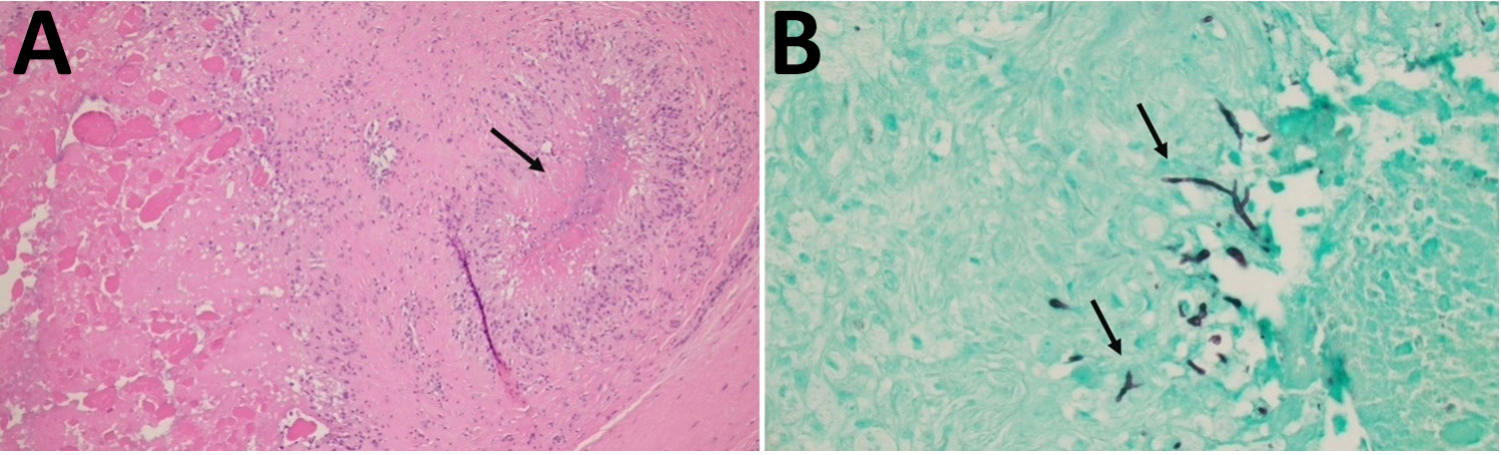
Images of stained tissue specimen from olecranon bursa of case-patient 1 in study of chronic elbow nodules caused by *Fomitiporella* fungus in immunocompetent patients, United States. A) Hematoxylin and eosin staining shows multiple large expansile back-to-back necrotizing granulomas surrounded by epithelioid histiocytes (arrow). Original magnification ×100. B) Grocott methenamine-silver staining shows many fungal hyphae visible throughout the granulomas (arrows). Original magnification ×400.

Histopathologic examination of the removed tissue revealed multiple large expansile granulomas bordered by epithelioid histiocytes. Gram and acid-fast stains were negative; alcian blue/periodic acid–Schiff staining showed focal dermal mucin and abundant fungal hyphae, and Grocott methenamine-silver stain highlighted numerous hyphae within granulomatous areas (Figure 1). Culture was not ordered. A formalin-fixed, paraffin-embedded (FFPE) tissue block was submitted to the University of Washington (UW) Molecular Microbiology Laboratory (Seattle, WA, USA) for molecular diagnosis.

Broad-range fungal PCR (*10*) targeting the 28S rRNA gene yielded a 610-bp sequence (GenBank accession no. PX488794) with 98.2% identity to *Fomitiporella austroasiana* (accession no. MG657323) and 98.03% identity to 2 *F. austroasiana* type strain sequences (accession nos. MG657320 and NG_060440). We corroborated the result by comparing a 316-bp internal transcribed spacer (ITS1) sequence (accession no. PX596867) to the same strains, which showed 98.2% identity to *F. austroasiana* (accession no. MG657323) and 90.77% identity to 2 type strain sequences (GenBank accession nos. MG657328 and NR_158435), establishing a molecular diagnosis of *Fomitiporella* species related to *F. austroasiana*. The patient received no antifungal therapy and remained well without recurrence.

**Figure 2:**
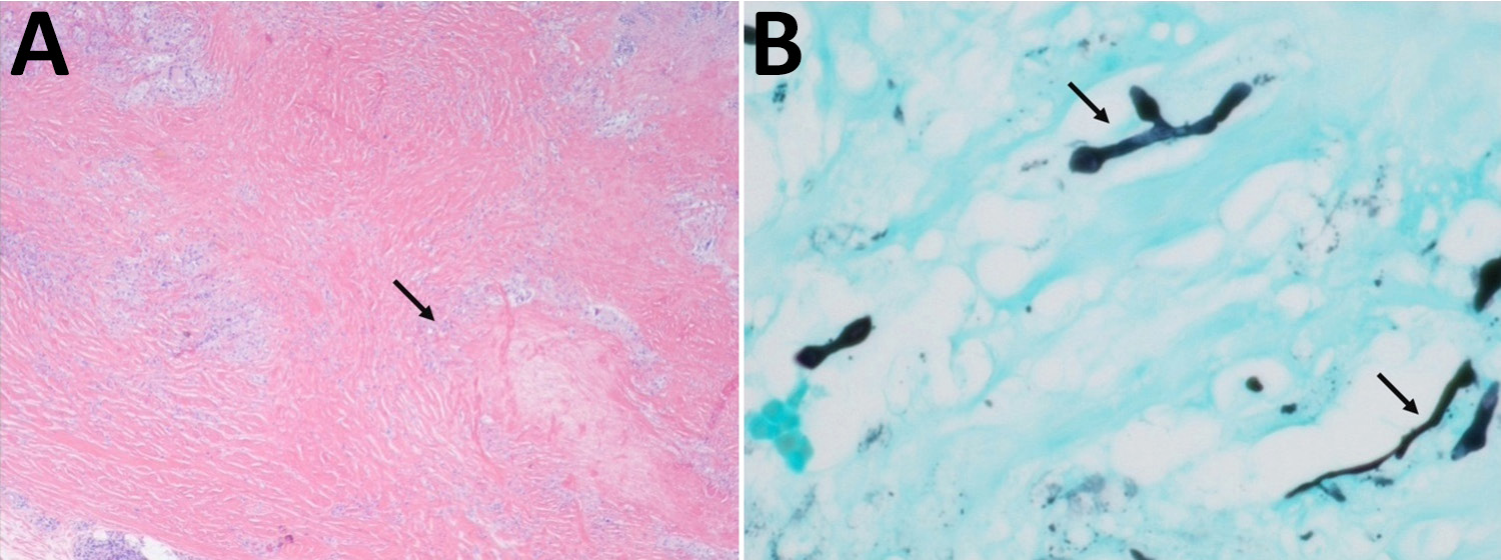
Images of stained tissue specimen from olecranon bursa of case-patient 2 in study of chronic elbow nodules caused by *Fomitiporella* fungus in immunocompetent patients, United States. A) Hematoxylin and eosin staining shows sclerotic granuloma (arrow). Original magnification ×20. B) Grocott methenamine-silver staining shows many fungal hyphae visible throughout the granulomas (arrows). Original magnification ×400.

Case 2 was in a 43-year-old man in Washington who immigrated from Mexico in 2021. He sought care in 2025 for a painful, slowly enlarging nodule over his left olecranon bursa that had persisted for >5 years. He had no systemic symptoms or history of immunodeficiency. Surgical excision of the bursa was performed, and a specimen was submitted for histopathology. Immunohistochemical staining showed no infiltrative cytokeratin AE1/AE3 or CD34-positive cells. Although acid-fast staining results were negative, GMS stain revealed scattered fungal hyphae along the periphery of palisading granulomas (Figure 2). Histopathologic features were consistent with subcutaneous fungal infection. An FFPE tissue block was sent to the UW Molecular Microbiology Laboratory for broad-range fungal PCR. 

A 608-bp 28S rRNA gene sequence from the specimen (GenBank accession no. PX488793) displayed 98.8% identity to *F. micropora* (accession no. MG806099). Results of 327-bp ITS sequencing of the reference strain (accession no. PX596866) provided orthologous support, showing 91.25% identity to *F. micropora* (accession no. KX181296). We established the molecular diagnosis of *Fomitiporella* species related to *F. micropora* on the basis of those results. The patient received no antifungal therapy and remained well without recurrence.

The 2 cases presented here implicate *Fomitiporella* spp. as human pathogens. In both cases, the DNA sequences identified exhibited sufficient sequence diversity from previously classified *Fomitiporella* spp. that they likely represent previously undescribed species in the genus. Case 1 involved a *F*. *austroasiana*–like species; case 2 involved a *F*. *micropora* lineage. Human infections attributed to *Fomitiporella* spp. are rarely reported in the literature; an infection involving a *F*. *micropora* lineage was previously identified by the UW Molecular Microbiology Laboratory in an immunocompromised patient in New York in 2025 (*11*). The cases we report demonstrate that *Fomitiporella* can cause disease in otherwise healthy hosts.

Clinicians initially considered chronic aseptic olecranon bursitis in both cases; histopathologic examination unexpectedly revealed fungal hyphae, prompting consultation with the infectious diseases service. Each of the 3 reported *Fomitiporella* infections have manifested as long-standing, localized cutaneous or subcutaneous lesions: 1 on the lower extremity (*11*) and 2, the cases we describe, involving the olecranon. That pattern suggests superficial soft tissue as a favored niche, possibly after environmental inoculation via unnoticed trauma or occupational exposure; for 1 patient, documentation indicated long-term employment in equipment assembly. In other implantation mycoses, patients often do not recall a specific traumatic event. Of note, all 3 reported infections have occurred in persons who immigrated from tropical or subtropical regions (Liberia, Mexico, and Trinidad), where wood-decaying *Fomitiporella* spp. are endemic (*12*), suggesting that patients acquired the infection before immigration. However, the apparent geographic distribution might partly reflect where environmental sampling for those fungi has been conducted.

Both of the case-patients we report were treated with surgical excision and no antifungal therapy. Although long-term follow-up data were not available, the clinical course in those cases and the previously reported case (*11*) suggested that surgical excision might be sufficient for localized *Fomitiporella* infection and with antifungal therapies reserved for incomplete excision, immunosuppressed patients, or evidence of dissemination. In cases where antifungal treatment is warranted, the limited evidence suggests that infections caused by filamentous basidiomycetes usually respond well to azoles other than fluconazole (*7*,*13*).

## Conclusions

Filamentous basidiomycetes are rarely implicated in human infection and are often misidentified or overlooked. Awareness of *Fomitiporella* is important for pathologists who encounter fungal hyphae in granulomatous lesions without a definitive culture result. Molecular identification should be considered when cultures are sterile or yield nonsporulating hyaline molds. Reporting additional *Fomitiporella* cases will improve understanding of epidemiology, disease spectrum, and treatment strategies.

*Fomitiporella* spp. infection, although exceedingly rare, can cause chronic localized lesions in both immunocompetent and immunocompromised hosts. Complete surgical excision offers excellent outcomes without antifungal therapy in most patients. Broad-range fungal PCR can identify molds and yeasts and some nonfungal eukaryotic pathogens such as oomycetes, amoebae, and parasites (*14*,*15*); therefore, it is a valuable tool for diagnosing unusual or unexpected infections, even in cases in which material is not available for microbial culture. Clinical and laboratory awareness of the pathogenic potential of *Fomitiporella* spp. will contribute to increased recognition and prevent misdiagnosis of such potentially emerging pathogens.
